# Pupil Dilation With Intra-Cameral Lidocaine Versus Topical Midriatics During Phacoemulsification

**DOI:** 10.5539/gjhs.v6n7p8

**Published:** 2014-09-18

**Authors:** Vafa Samarai, Negar Haghighi, N. Sharifi

**Affiliations:** 1 Department of Ophthalmology, Imam Hospital, Urmia University of Medical sciences, Urmia, Iran; 2 Urmia University of Medical Sciences, Urmia, Iran

**Keywords:** cyclopentolate, epinephrine, intra-cameral, midriatic, pseudo-exfoliation

## Abstract

**Purpose:**

To evaluate pupil dilation with intra-cameral injection of preservative-free lidocaine 1% (ICL) versus topical eye midriatics during phacoemulsification.

**Methods:**

This case-control study included 40 patients with similar bilateral senile cataract scheduled for phacoemulsification and intraocular lens (IOL) implantation. patient’s first eye received topical midriatic eye drops as control group and next eye operated by intra cameral preservative free lidocaine 1% without any preoperative or intraoperative midriatics. We did not add epinephrine to the irrigating solution in either group. The first eyes received 3 drops of cyclopentolate 1% and tropicamide1% each 5 minutes, with first dose 60 minutes before surgery. The horizontal pupil diameter was measured before and after pupil dilation using the same caliper with operation microscope total surgical time was recorded in both groups.

**Results:**

Patients included 20 male and 20 female with mean age of 72 and 70.9 years old .4 patients were diabetic and 11 cases had pseudo-exfoliation. Pupil diameter increased in both case and control groups significantly (P value<0.0) but the difference between mean increase in pupil size wasn’t significantly different. Mean increase in pupil size was significantly greater in patients without pseudo-exfoliation (4.10 mm vs 3.85 mm, independent t test, P<0.05). There was no significant difference between diabetic and non- diabetic patients regarding of pre- and post-injection diameter of the pupil.

**Conclusion:**

Intra-cameral preservative-free lidocaine 1% supply adequate midriasis during cataract surgery by itself.

## 1. Introduction

Phacoemulsification starts with pupil dilation in order to good visualization of opaque lens. It can be achieved by topical midriatic drops prior to surgery and/or intra cameral administration of epinephrine. Cyclopantolate, tropicamide and phenylephrine are the most popular midriatic drops. These agents have some disadvantages such as slow onset of midriasis (Aminollah, 2008; Maria et al., 2000) cardiovascular side effects especially in high risk groups like elderly patients with hypertension (Aminollah, 2008; Maria et al., 2000; Weller, Pham, & Häberle, 2002) tendencies to wear off during the surgery1, toxic eye and systemic effects (Maresová, 2009; Myers, 2009). Lidocaine is an antiarrhythmic agent and it’s known for its anesthetic effect. The efficacy profile of lidocaine as a local anesthetic is characterized by a rapid onset of action and intermediate duration of efficacy. Therefore, lidocaine is suitable for infiltration, block and surface anesthesia (Cepeda, 2010). Adverse drug reactions (ADRs) are rare when lidocaine is used as a local anesthetic and is administered correctly. Most ADRs associated with lidocaine for anesthesia relate to administration technique (resulting in systemic exposure) or pharmacological effects of anesthesia, but allergic reactions only rarely occur (Jackson, 1994). Intra-cameral lidocaine HCl appears to be safe for the average phacoemulsification (Maria et al., 2000; Weller et al., 2002). Intra-cameral lidocaine acts by Uveal anaesthesia and decrease sensitivity to microscope light. Multiple studies have showed that the supplement of intra-cameral preservative-free lidocaine 1%. To anesthetic drops provides increased patient comfort, particularly during AC deepening and iris manipulation. Preservative-free lidocaine 1% is well tolerated by the corneal endothelium (Khokhar & Rahim, 2010). There is no significant difference in endothelial cell loss between phacoemulsification with prebulbar anesthesia and combined tetracaine 1% and intra-cameral lidocaine (Lundberg, 2009; Nikeghbali, 2007; Cionni, 2003; Benatar-Haserfaty, 2004; B. Anders & E. Anders, 2004; Adcock et al., 2003; Ogut et al., 1996).

## 2. Patients and Method

A prospective interventional case control study was performed to compare the pupil dilation effect of intra cameral lidocaine alone versus topical midriatic eye drops during cataract surgery. The study was performed according to the principles of the Declaration of Helsinki and all patients signed an informed consent. Local review boards of ethics committee approved study. 40 patients with bilateral senile cataract scheduled for phacoemulsification and intraocular lens (IOL) implantation. Only patients with similar bilateral lens opacity were included. Patients with intumescent cataract and previous ocular surgery or laser procedures, iris abnormalities, and patients using any topical ocular medications (except artificial tears) were excluded. We matched the nuclear color and opalescence of the patient′s lenses between two eyes on the slit-lamp examination with the standard photographs of Lens Opacities Classification System III (LOCS III). The order of surgery was based on visual acuity. In cases of similar visual acuity, the patient was asked about which eye has worse vision. If the answer was the same, one of them was operated first randomly.

.All surgeries were carried out by the same surgeon (V.S.) under topical anesthesia in an outpatient setting. The nucleus was removed by Legacy phacoemulsification machine (Alcon, TX). Using the stop and chop technique. The cortex was removed with bimanual irrigation & aspiration, followed by polishing the posterior capsule. We used hydroxypropyl methylcellulose 2% (Bausch and Lomb, Waterford, Ireland) as a viscoelastic agent and balanced salt solution (BSS) as irrigation solution (without added epinephrine). All patients received three drops of tetracaine 1% started five minutes before surgery. After creation of a side-port incision 0.2 ml of preservative-free lidocaine 1% was injected into the anterior chamber. In both groups, horizontal pupil diameter was measured with a caliper using the operating microscope in the supine position. Light intensity of the microscope was the same during measurements and surgery Total surgical time was measured from the time of first incision to the end of operation when the blepharostat was removed. Phacoemulisification with IOL implantation was done on the first eye using topical midriatic eye drops (Tropicamide1%, cyclopentolate 1%,) one drop each 5 minute started 45 minutes prior to surgery (3 doses) as control group (group1), horizontal diameter of the pupil was measured with a caliper under the operating microscope light just before starting midriatic drops. Measurement was repeated just before surgery. The second eye was operated approximately one month later (28-35 days). This time only intra cameral injection of preservative free lidocaine 1% was used for pupil dilation. Pupil diameter was measured just before surgery and 90 seconds after intra-cameral injection. This method was performed only To minimize the cofounder factors like age, sex, underlying diseases, genetic factors, drug sensitivity, pupil horizontal diameter, general condition between case and control groups.

## 3. Results

With the mean age of 72 and 70.9 years, respectively. All patients had grade 3 or more lens opacity in locs III grading system. Four of the patients (2 female and 2 male) had diabetes mellitus (DM) and 11 patients (5 male and 6 female) had Pseudo exfoliation (PXF). Table 1 shows patient characteristics. Average pupil diameter increase in both groups which wasn’t significantly different (P value: 0.15). TABLE 2-4 demonstrates subgroup analysis: average pupil diameter increase was significantly higher in patients of pure cataract than in patients with simultaneous PXF in both groups. Difference between diabetic and non-diabetic subgroups was not significant. There is no significant difference in pupillary response between men and women in both groups (Table 4). Total operation time did not change significantly in this study between two groups.

**Table 1:** Group characteristics

Parameter	Topical midriatic	Intra-cameral lidocaine	P value
Number of eyes	40	40	
Male	20	20	
Female	20	20	
Mean age(y)	71.45	71.45	
Mean surgical time(min)	12.705	12.700	0.88
Mean baseline pupil diameter before drug use(mm)	2.74	2.71	0.72
Mean baseline pupil diameter after drug use(mm)	6.67	6.54	0.47
Mean increase in pupil diameter(mm)	4.10 -+0.07	3.93+- 0.08	0.15

**Table 2:** Subgroup data in topical midraiatic group (TM)

Patients	Average pupil diameter before topical midriatics(mm)	Average pupil diameter after topical midriatics(mm)	Average pupil diameter increase after topical midriatics(mm)
Only cataract	3.00	6.99	4.06
Cataract+ PXF	2.49	6.40	3.90
Cataract+ DM	2.75	6.62	4.12

**Table 3:** Subgroup data in intra-cameral lidocaine group (ICL)

Patients	Average pupil diameter before ICL(mm)	Average pupil diameter after ICL(mm)	Average pupil diameter increase After ICL
Only cataract	2.85	6.96	4.10
Cataract+ PXF	2.52	6.18	3.85
Cataract+ DM	2.77	6.50	3.88

**Table 4:** gender specific average pupil diameter increase after pupil dilation

	Average pupil diameter increase after ICL(mm) Case group	Average pupil diameter increase after topical midriatics (mm) Control group
Female	4.13	4.22
Male	3.90	3.80
Total	3.92	4.01

**Table 5:** Total operation time

Patients	Total operation time in TM group (minute)	Total operation time in ICL group (minute)
Cataract- PXF	12.40	12.51
Cataract + PXF	13	12.9

## 4. Discussion

Topical anesthesia has gained popularity for modern cataract surgery in recent years. Tetracaine1% by itself leads to a good analgesia in most cases. However some of the patients experience pain during surgery. Combined intra-cameral lidocaine and topical Tetracaine1% has improved the efficacy of the topical anesthesia significantly. Topical midriatic drops have been used to dilate. The pupil during cataract sugary for many years, but these agents has some disadvantages. This study was designed to evaluate safety of intra-cameral lido as an alternative to topical midriatic drops. Recently lidocaine is known for its antiarrhythmic, anti-convulsive and antitussive effects, too (Adcock et al., 2003). Lidocine was applied in cataract surgery as antalgic agent since many years ago. It’s simultaneous midriatic effect addition to annoying side effect of traditional midriatic eye drops makes it a favorite subject for clinicians to study about. ICL in Maresova ‘s study is mentioned to be a safe midriatic, also its effect as an midriatic agent is adequate and long standing enough for cataract surgery (Maresova, 2009). Coinni studied ICL as a single midriatic agent in cataract surgeries and reported its advantages like faster function due to returning pupil size to normal size quickly and diminished rate of punctuate keratopathy. Midriatic eye drops complications were also omitted in this method (Cionni, 2003). In Behndig study includes ICL and phenylephrine for midriases in cataract surgery, any increase in complication rates or operation time was not reported. Good subject surgery performance also was an advantage of this method. Nikeghbali studied ICL and topical eye drops in an interventional study in which ICL was known as an safe and effective alternative to those drops in Phacoemulsification (Nikeghbali, 2007). In two similar study conducted by Lundberg and Myers two midriatic solution including lidocaine and phenylephrine used, in which Cyclopantolate was omitted from one of them this studies result illustrated that Cyclopantolate does not enhance midriasis in cataract surgery (Lundberg, 2009) and (Myers, 2009).

In present study we used two techniques of pupil dilation and compare them in effectiveness like other studies but we conducted them in 2 eyes of each patient, so any confounder factor between cases and controls was omitted. Also the results support previous studies. All patients were selected according to same inclusion and exclusion criteria at beginning of the study to preserve the main concordance between under study groups. Common underlying diseases in patients with cataract like diabetes and pseudo-exfoliation which could affect pupil and as a result, dilating it, were also studied, and as it is obvious, intra cameral lidocaine can be used in these groups as a safe alternative to topical midriatics too. In our study total surgery time didn’t have significant difference by two method but using ICL induced a rapid dilation prior to surgery and also pupil returns to it normal size faster. Additional studies about post-surgery short-term follow up are recommended to clear lidocaine’s effects on the ocular components or any probable complications.
